# Supplementary figures and images for: A field-based investigation of behavioural interactions between invasive green crab (Carcinus maenas), rock crab (Cancer irroratus), and American lobster (Homarus americanus) in southern Newfoundland
Source: PeerJ. 2020 Feb 12;8:e8444. doi: 10.7717/peerj.8444 (PMC7023827; doi:10.7717/peerj.8444)

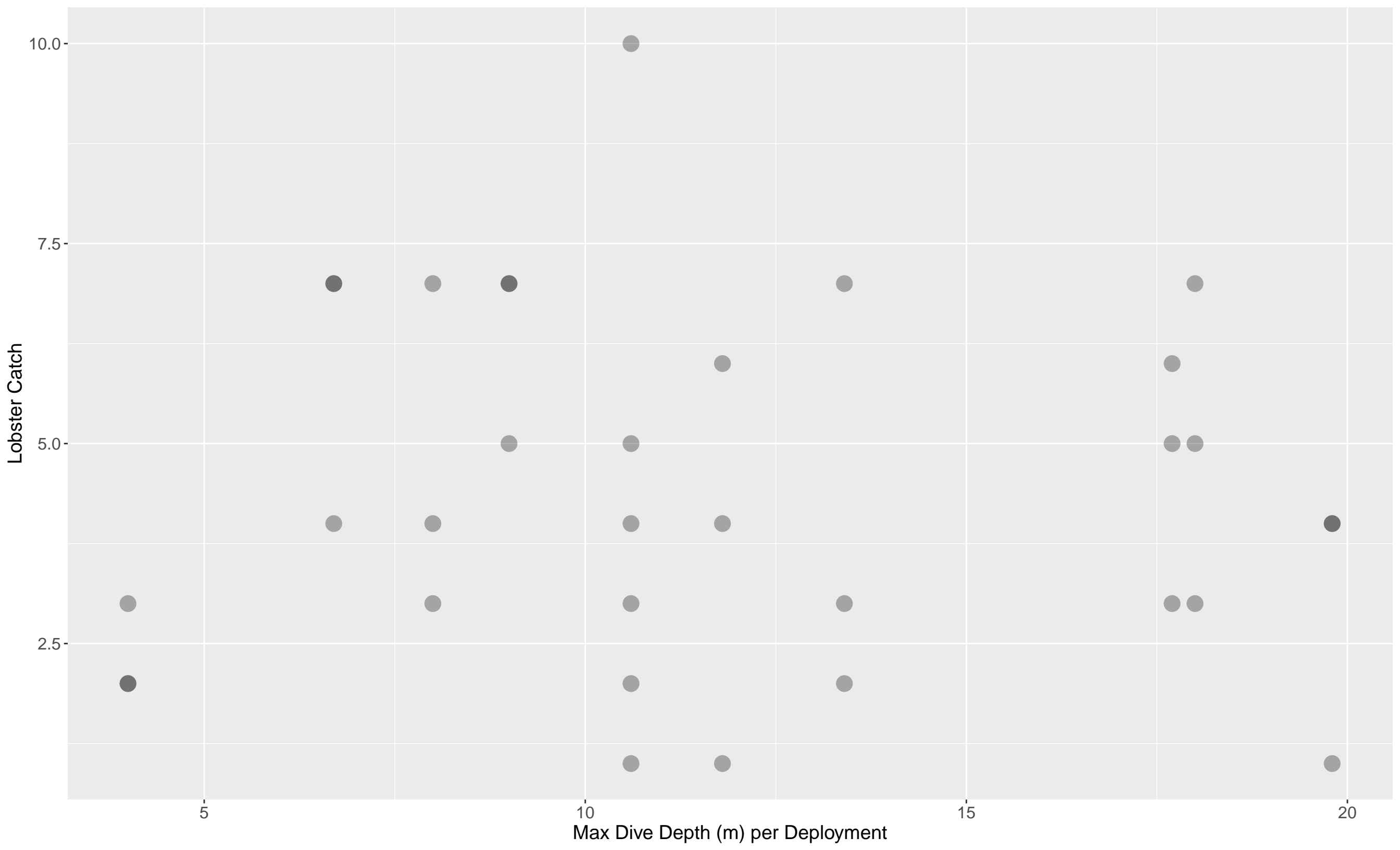

Supplement: Figure S1 [file peerj-08-8444-s001.pdf]

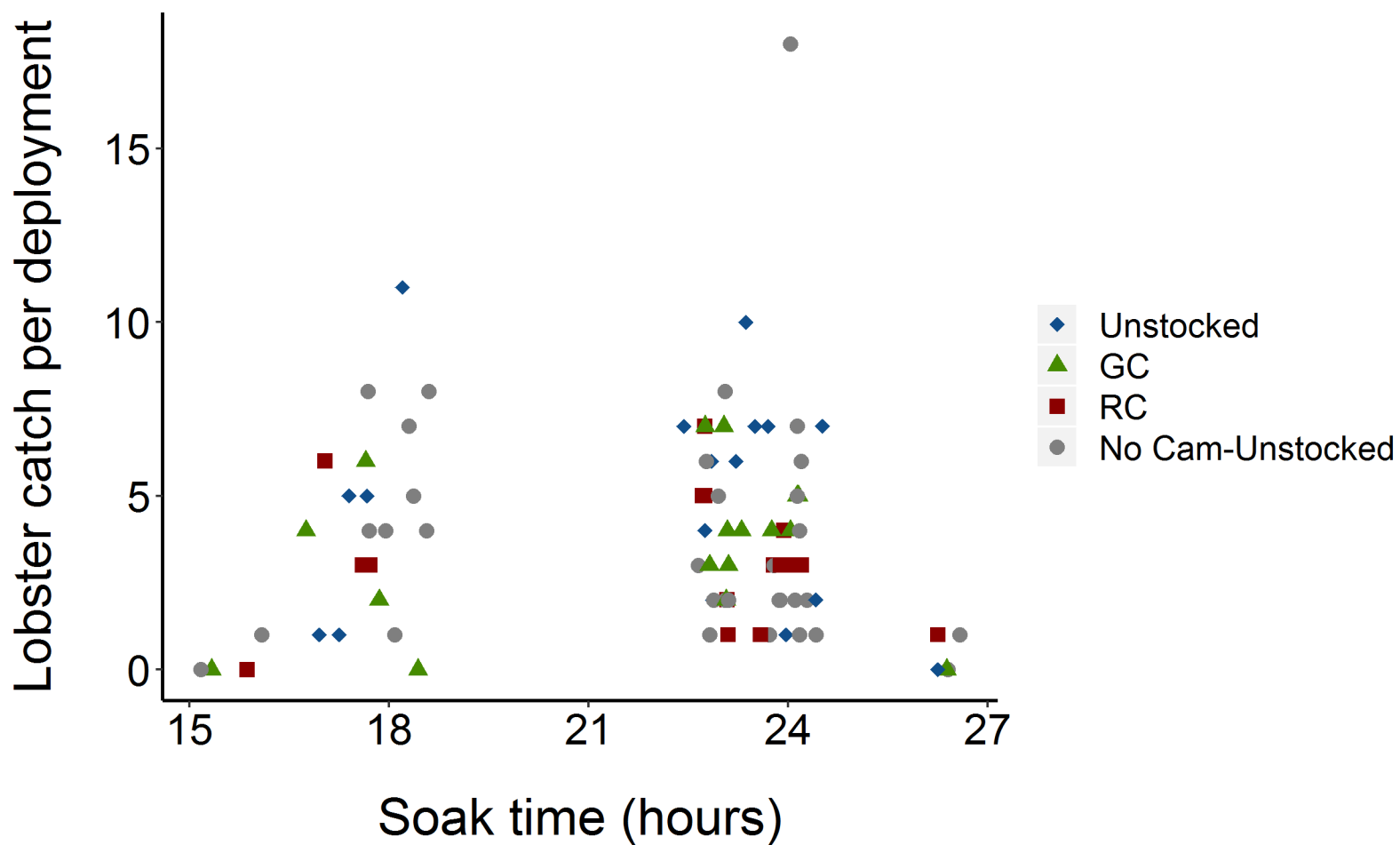

Supplement: Figure S2 [file peerj-08-8444-s002.pdf]

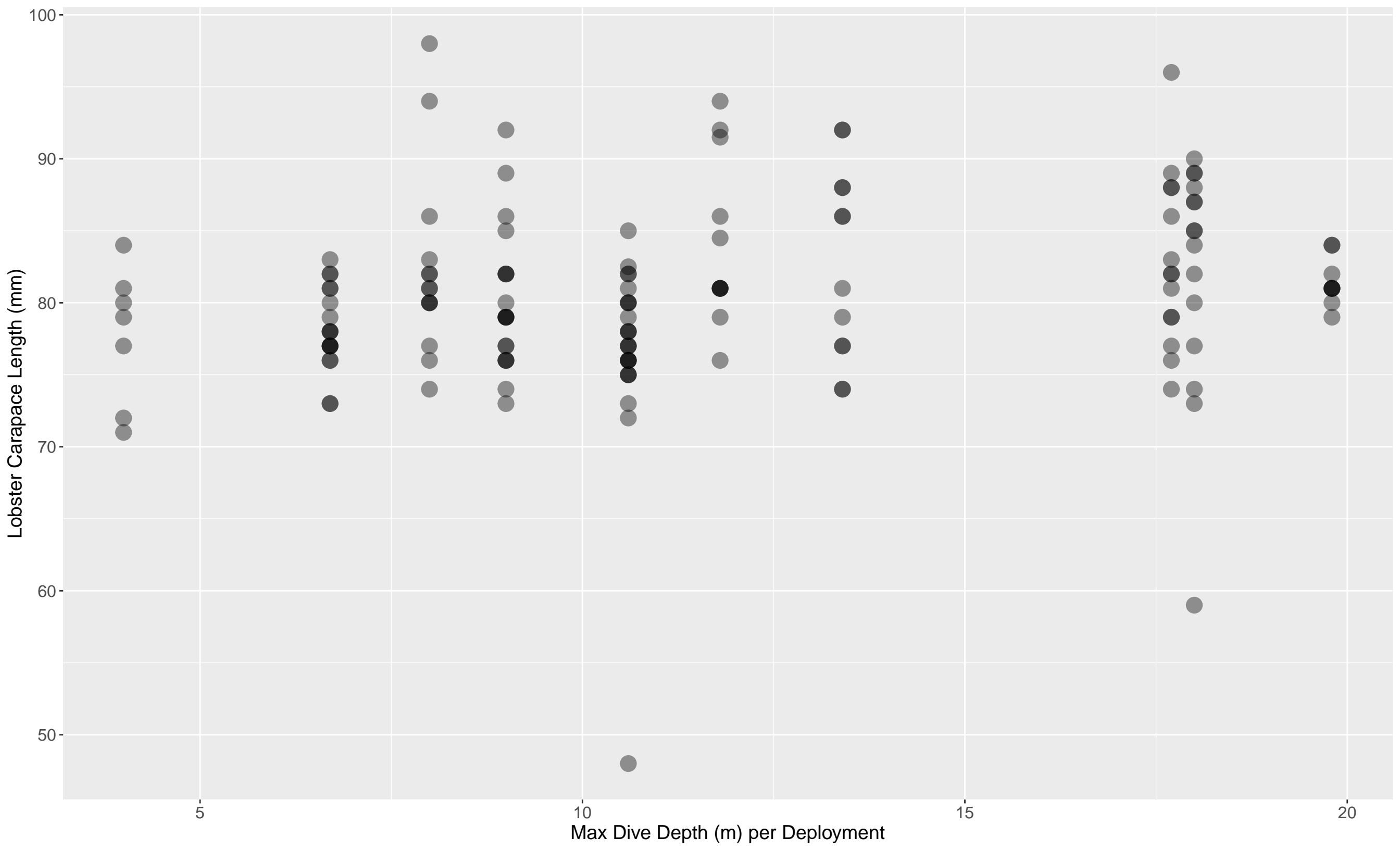

Supplement: Figure S3 [file peerj-08-8444-s003.pdf]

Duration of entries (minutes)

10.0  
1.0  
0.1

Unstocked

GC

RC

Pre-stocking condition

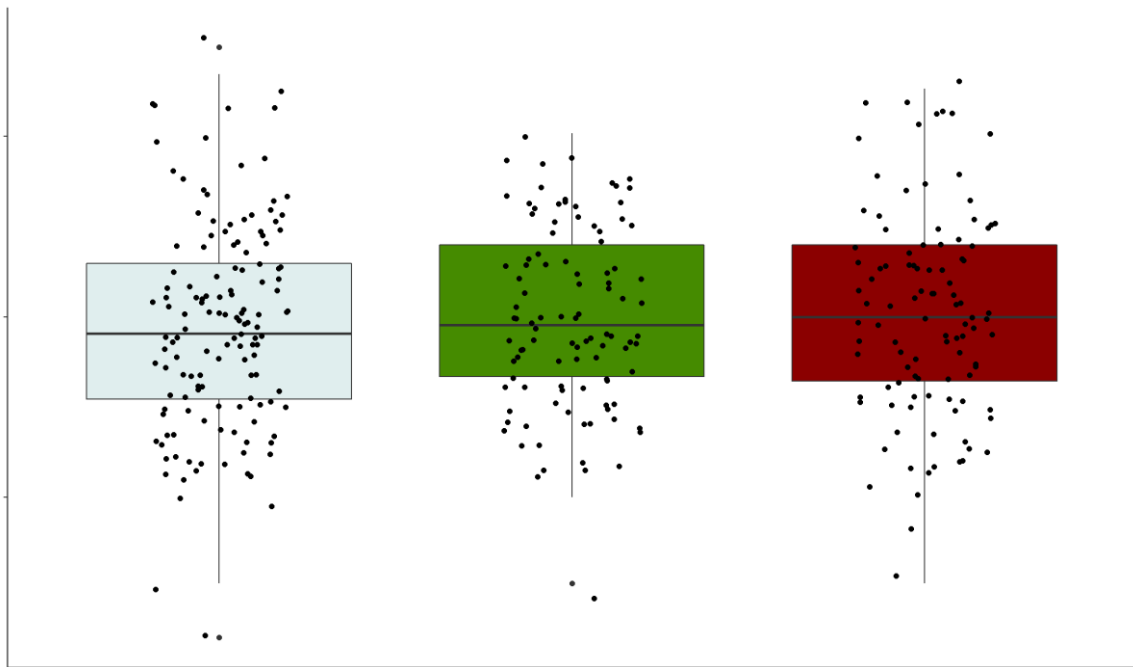

Supplement: Figure S4 — The horizontal lines indicate the medians for each trap pre-stocking condition. Each dot represents a single entry attempt. [file peerj-08-8444-s004.pdf]

Pre-stocking condition

Unstocked

RC

GC

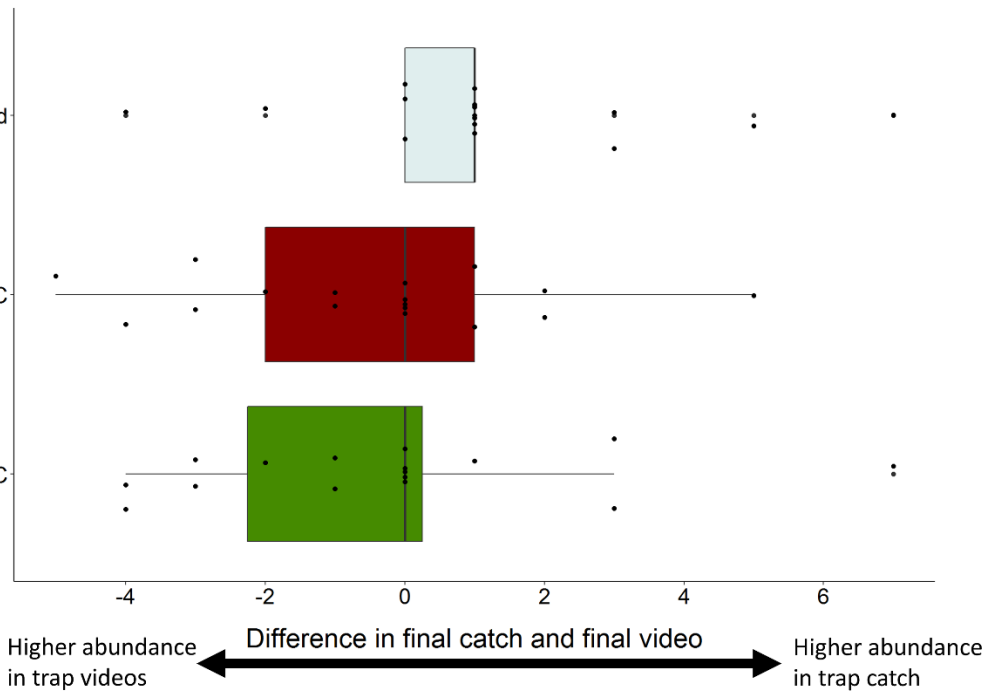

Supplement: Figure S5 [file peerj-08-8444-s005.pdf]

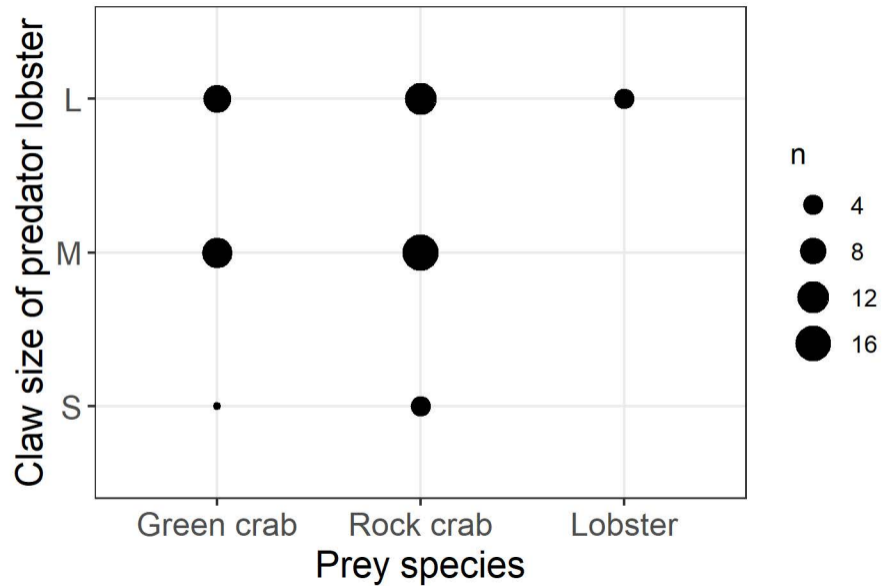

Supplement: Figure S6 — S indicates small lobster claw size (less than 6 cm); M indicates medium claw size (between 6 cm and 8 cm); L indicates large claw size (greater than 8 cm). [file peerj-08-8444-s006.pdf]

Lobster catch per deployment

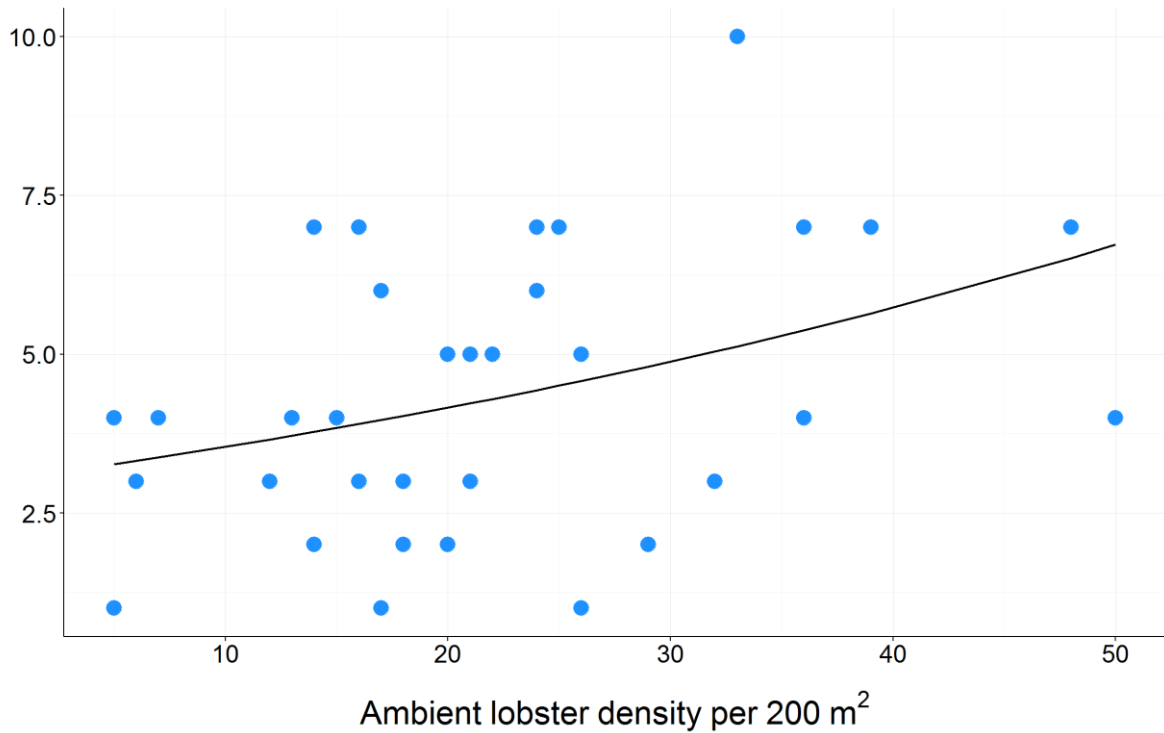

Supplement: Figure S7 [file peerj-08-8444-s007.pdf]
